# Supplementary material for: HIVprotI: an integrated web based platform for prediction and design of HIV proteins inhibitors
Source: J Cheminform. 2018 Mar 9;10:12. doi: 10.1186/s13321-018-0266-y (PMC5845081; doi:10.1186/s13321-018-0266-y)
Supplement: Supplementary file 2 — Additional file 2. Source code of HIVProtI web server. [file 13321_2018_266_MOESM2_ESM.zip › HIVprotI_Source-code/hivprotI/weka-3-6-6/doc/weka/core/pmml/MiningFieldMetaInfo.html]

MiningFieldMetaInfo


---


|  |  |  |
| --- | --- | --- |
| **Overview** \| **Package** \| **Class** \| **Tree** \| **Deprecated** \| **Index** \| **Help** \| **Weka's home** | |  |
| **PREV CLASS**   **NEXT CLASS** | **FRAMES**    **NO FRAMES**     **All Classes** |  |
| SUMMARY: NESTED \| FIELD \| CONSTR \| METHOD | DETAIL: FIELD \| CONSTR \| METHOD |  |


---


## weka.core.pmml Class MiningFieldMetaInfo

```
java.lang.Object
  weka.core.pmml.FieldMetaInfo
      weka.core.pmml.MiningFieldMetaInfo
```

**All Implemented Interfaces:**: java.io.Serializable

---

``` public class MiningFieldMetaInfo extends FieldMetaInfo implements java.io.Serializable ```

Class encapsulating information about a MiningField.

**Version:**
:   $Revision: 5562 $

**Author:**
:   Mark Hall (mhall{[at]}pentaho{[dot]}com)

**See Also:**: Serialized Form

---

**Nested Class Summary**

**Nested classes/interfaces inherited from class weka.core.pmml.FieldMetaInfo**

`FieldMetaInfo.Interval, FieldMetaInfo.Optype, FieldMetaInfo.Value`


**Constructor Summary**

`MiningFieldMetaInfo(org.w3c.dom.Element field)`
  
          Constructs a new MiningFieldMetaInfo object.


| **Method Summary** | |
| --- | --- |
| `double` | `applyMissingValueTreatment(double value)`             Apply the missing value treatment method for this field. |
| `double` | `applyOutlierTreatment(double value)`             Apply the outlier treatment method for this field. |
| `Attribute` | `getFieldAsAttribute()`             Return this mining field as an Attribute. |
| `weka.core.pmml.MiningFieldMetaInfo.Missing` | `getMissingValueTreatmentMethod()`             Get the missing value treatment method for this field. |
| `java.lang.String` | `getName()`             Get the name of this field. |
| `weka.core.pmml.MiningFieldMetaInfo.Outlier` | `getOutlierTreatmentMethod()`             Get the outlier treatment method used for this field. |
| `weka.core.pmml.MiningFieldMetaInfo.Usage` | `getUsageType()`             Get the usage type of this field. |
| `void` | `setIndex(int index)`             Set the index of this field in the mining schema Instances |
| `java.lang.String` | `toString()`             Return a textual representation of this MiningField. |

**Methods inherited from class weka.core.pmml.FieldMetaInfo**

`getFieldName, getOptype`

**Methods inherited from class java.lang.Object**

`equals, getClass, hashCode, notify, notifyAll, wait, wait, wait`

**Constructor Detail**

### MiningFieldMetaInfo

```
public MiningFieldMetaInfo(org.w3c.dom.Element field)
                    throws java.lang.Exception
```

:   Constructs a new MiningFieldMetaInfo object.

    **Parameters:**: `field` - the Element that contains the field information **Throws:**: `java.lang.Exception` - if there is a problem during construction


**Method Detail**

### getUsageType

```
public weka.core.pmml.MiningFieldMetaInfo.Usage getUsageType()
```

:   Get the usage type of this field.

    :   **Returns:**: the usage type of this field

---


### toString

```
public java.lang.String toString()
```

:   Return a textual representation of this MiningField.

    :   **Overrides:**: `toString` in class `java.lang.Object`
    :   **Returns:**: a String describing this mining field

---


### setIndex

```
public void setIndex(int index)
```

:   Set the index of this field in the mining schema Instances

    :   **Parameters:**: `index` - the index of the attribute in the mining schema Instances that this field represents

---


### getName

```
public java.lang.String getName()
```

:   Get the name of this field.

    :   **Returns:**: the name of this field

---


### getOutlierTreatmentMethod

```
public weka.core.pmml.MiningFieldMetaInfo.Outlier getOutlierTreatmentMethod()
```

:   Get the outlier treatment method used for this field.

    :   **Returns:**: the outlier treatment method

---


### getMissingValueTreatmentMethod

```
public weka.core.pmml.MiningFieldMetaInfo.Missing getMissingValueTreatmentMethod()
```

:   Get the missing value treatment method for this field.

    :   **Returns:**: the missing value treatment method

---


### applyMissingValueTreatment

```
public double applyMissingValueTreatment(double value)
                                  throws java.lang.Exception
```

:   Apply the missing value treatment method for this field.

    :   **Parameters:**: `value` - the incoming value to apply the treatment to **Returns:**: the value after applying the missing value treatment (if any) **Throws:**: `java.lang.Exception` - if there is a problem

---


### applyOutlierTreatment

```
public double applyOutlierTreatment(double value)
                             throws java.lang.Exception
```

:   Apply the outlier treatment method for this field.

    :   **Parameters:**: `value` - the incoming value to apply the treatment to **Returns:**: the value after applying the treatment (if any) **Throws:**: `java.lang.Exception` - if there is a problem

---


### getFieldAsAttribute

```
public Attribute getFieldAsAttribute()
```

:   Return this mining field as an Attribute.

    :   **Specified by:**: `getFieldAsAttribute` in class `FieldMetaInfo`
    :   **Returns:**: an Attribute for this field.


---


|  |  |  |
| --- | --- | --- |
| **Overview** \| **Package** \| **Class** \| **Tree** \| **Deprecated** \| **Index** \| **Help** \| **Weka's home** | |  |
| **PREV CLASS**   **NEXT CLASS** | **FRAMES**    **NO FRAMES**     **All Classes** |  |
| SUMMARY: NESTED \| FIELD \| CONSTR \| METHOD | DETAIL: FIELD \| CONSTR \| METHOD |  |


---
